# Antioxidative and Anti-Inflammatory Protective Effects of Fucoxanthin against Paracetamol-Induced Hepatotoxicity in Rats

**DOI:** 10.3390/md21110592

**Published:** 2023-11-14

**Authors:** Maimonah Fuad Koshak, Mahmoud Zaki El-Readi, Mohamed Elzubier Elzubier, Bassem Refaat, Riyad Adnan Almaimani, Shakir Idris, Mohammad Althubiti, Hiba Saeed Al-Amodi, Safaa Yehia Eid

**Affiliations:** 1 Department of Biochemistry, Faculty of Medicine, Umm Al-Qura University, Al Abdeyah, Makkah 24381, Saudi Arabia; 2 Laboratory of Clinical Chemistry, King Salman Armed Forces Hospital, Tabuk 47512, Saudi Arabia; 3 Laboratory Medicine Department, Faculty of Applied Medical Sciences, Umm Al-Qura University, Al Abdeyah, P.O. Box 7607, Makkah 24381, Saudi Arabia

**Keywords:** paracetamol, acetaminophen, liver injury, fucoxanthin, inflammation, oxidative stress

## Abstract

Paracetamol or acetaminophen (PAC) is a commonly used analgesic and antipyretic drug. It has been shown that overdoses beyond the therapeutic range can cause hepatotoxicity and acute liver injury. The most common cause of drug-induced liver injury (DILI) in Saudi Arabia and worldwide is paracetamol overdose. Fucoxanthin (FUC) is an allenic carotenoid that is found in edible brown seaweeds, and it has antioxidant and anti-inflammatory effects. Several studies have shown the potential therapeutic effects of FUC in diabetes, cancers, and inflammatory disorders. This study aims to investigate the protective effect of FUC against PAC-induced acute liver injury in rats. FUC was administered (100, 200, and 500 mg/kg, p.o.) for 7 days, and then the liver injury was induced by the administration of PAC (2000 mg/kg, oral). Blood and liver tissue samples were collected from PAC-positive untreated, treated, and negative control rats. Biochemical and inflammatory parameters in the blood were measured. In addition, RT-PCR, Western blotting, and immunohistochemistry were performed for liver tissue. The serum levels of liver biomarkers (ALT, AST, and ALP) increased after PAC-induced liver toxicity; FUC-treated rats showed lower levels compared to the positive control. There was an increase in the expression of TNF-α, IL-1, IL-6, NF-kB, INF-γ, and iNOS and a decrease in IL-10, IL-22, and IL-10R expression after the FUC treatment of injured liver rats. For the hepatic inflammation and PAC-toxicity-induced oxidative stress genes and proteins, FUC-treated rats (100, 200, and 500 mg/kg) showed a reduction in the expression of oxidative stress genes. These results showed that FUC protected the liver against PAC-induced injury through antioxidant and anti-inflammatory actions. However, further clinical studies are required to confirm the findings.

## 1. Introduction

The liver plays a crucial role in the homeostasis maintenance of the human body. Liver damage is linked to oxidative stress and the immune system’s inflammatory response [1]. Detoxification, metabolism, protein synthesis, host defense, and immunological response are just some of the many essential functions maintained by the liver [1]. Acute liver failure is characterized by severe liver dysfunction manifested by coagulopathy, jaundice, and encephalopathy, typically in the absence of underlying liver disease [2]. Liver disease continues to be the leading cause of morbidity and mortality worldwide. Steatohepatitis, fibrous, and cirrhosis are among the types of liver damage associated with liver disease, which is a multifactorial disease with a complex pathophysiology [3].

In developed countries, the incidence of acute liver failure (also known as fulminant hepatic failure) is approximately 10 per one million individuals annually [2], with over 2000 cases diagnosed annually in the United States [4]. While viral hepatitis is one of the leading causes of acute liver failure around the world [2], drug-induced liver injury (DILI) is another cause of liver damage [4]. According to reports, DILI is the cause of half of all cases of acute liver injury [5]. According to the United States Acute Liver Failure Study Group’s data, acetaminophen is the primary causative agent for acute liver failure (ALF), accounting for 45.8% of cases. Non-acetaminophen drug-induced liver injury (DILI) follows with a prevalence of 11%, while isoniazid (INH) is the top cause of DILI subsequently, contributing to 18.8% of cases [6].

Paracetamol (PAC), alternatively referred to as acetaminophen or N-acetyl-ρ-aminophenol, is a frequently used antipyretic drug. It is recognized that elevated blood concentrations of paracetamol beyond the therapeutic threshold can result in hepatotoxicity [7].

The Food and Drug Administration (FDA) considered it safe to ingest a maximum dose of 4000 mg within a 24-h period due to the availability of this medication as an over-the-counter product and its broad therapeutic range [8,9]. However, individuals with pre-existing liver disease or chronic alcohol consumption are advised by practitioners to limit their intake to 2000 mg or less [9,10].

Intentional or accidental overdose of PAC is the leading cause of drug-induced liver injury (DILI) with symptoms of PAC overdose that include nausea, vomiting, abdominal pain, and jaundice. In severe cases, liver failure can occur, which can lead to coma and death, especially among children [10,11]. However, the underlying cause of its toxicity has been unclear.

It has been reported that hepatocytes convert PAC into non-toxic metabolites via microsomal cytochrome P450 (CYP450). This metabolism pathway via CYP450, specifically cytochrome P450 2E1 (CYP2E1), produces reactive oxygen species [12], originally believed to be the ultimate cause of liver injury in PAC overdose. Mitochondrial dysfunction has been credited as the primary source of free radicals and oxidative stress in PAC-induced hepatotoxicity [13]. Mitochondrial dysfunction begins with the formation of drug–protein adducts between N-acetyl-p-benzoquinone imine (NAPQI), a reactive non-toxic PAC metabolite, and associated mitochondrial proteins in the electron transport chain [14].

In the liver, PAC undergoes a molecular transformation that allows it to create stable, covalent bonds with cysteine residues in target proteins. However, the involvement of acetaminophen in liver failure is not fully explained by these covalent binding processes alone [15]. In times of stress, glutathione forms conjugates with cysteine residues in order to prevent them from oxidative degradation. According to reports, there is evidence indicating that PAC and its metabolites exhibit a dose-dependent capacity to induce glutathionylation [16]. The alteration affects proteins involved in mitochondrial fuel intake and energy production, leading to metabolic dysfunction and other consequences connected to PAC toxicity including oxidative stress and inflammation. This finding helps explain the medication’s toxicity at high dosages, especially among enzymes that are affected by PAC use without binding directly to the drug or its metabolites [16].

Additionally, an increase in the activity of mitochondrial complex I, a known site of free radical generation [17], occurs with PAC toxicity, and the level of activity was found to correlate with the degree of liver injury [14]. Oxidative stress and an out-of-control inflammatory response are two potential outcomes of excessive NAPQI production. The role of oxidative stress in PAC-induced organ injury is essential. The overproduction of reactive oxygen species (ROS) is associated with an increased risk of inflammatory response and cell death [18].

The antioxidant glutathione precursor N-acetyl cysteine (NAC) was used to avoid liver damage due to its well-established derivative and antidote status. Due to its hydrophilia and dose dependence, N-acetyl cysteine reduces PAC inflammation and toxicity but has adverse effects. Multiple dosages are needed to reverse PAC toxicity due to its short half-life. Neurotoxicity, hepatotoxicity, and anaphylactoid responses result from long-term use. After prolonged use, the process reverses, causing ROS-induced oxidative stress and liver damage [19]. The identification of a novel pharmaceutical agent possessing both preventative and therapeutic properties for liver injury has emerged as a pressing goal.

Liver injury can be prevented or reduced using natural products, such as carotenoids [20,21]. Fucoxanthin (FUC), an allenic carotenoid found in edible brown seaweeds, is a powerful antioxidant and anti-inflammatory. Several studies have demonstrated that FUC has anti-obesity, anti-tumor, anti-diabetic, antioxidant, anti-inflammatory, cardiovascular, and cerebrovascular properties [22].

As a result, FUC has the potential to be used in the prevention and treatment of chronic diseases. Even though FUC has a wide range of medicinal and nutritional properties, it has not been investigated previously in PAC-induced liver injury, so there is a rationality for testing FUC as protective agent in liver toxicity in vivo. In this study, the potential protective effect of FUC was examined against PAC-induced hepatic injury (PILI).

## 2. Results

### 2.1. Fucoxanthin Reduced Liver Serum Biomarkers in PILI

**Figure 1:**
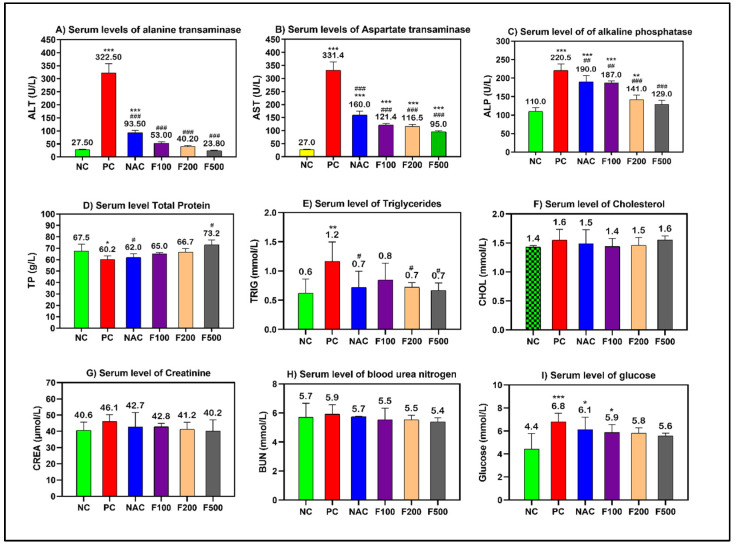
Effects of FUC on the serum levels of (**A**) alanine aminotransferase (ALT), (**B**) aspartate aminotransferase (AST), (**C**) alkaline phosphatase (ALP), (**D**) total protein (TP), (**E**) triglycerides, (**F**) cholesterol, (**G**) creatinine, (**H**) BUN, and (**I**) glucose in the rats with toxicity induced by PAC. The treated groups with different doses of FUC (F100 = 100 mg/kg, F200 = 200 mg/kg, and F500 = 500 mg/kg) and NAC groups were compared to the rats with liver injury or positive control rats (PC) and healthy rats (NC). The data are expressed as mean ± SD of the fold change. *, **, and *** indicate *p* < 0.05, *p* < 0.01, and *p* < 0.001, comparing all rat groups to healthy, non-treated, negative control (NC) rats; ^#^, ^##^, and ^###^ indicate *p* < 0.05, *p* < 0.01, and *p* < 0.001, comparing all rat groups to the rats with liver injury or positive control rats (PC), respectively.

Using a sub-lethal dose of paracetamol (2 g/kg) caused liver damage in the rats, which was demonstrated by a significant increase in the serum levels of transaminases (AST and ALT), alkaline phosphatase (ALP), and triglycerides (TP). Paracetamol increased liver enzymes in the rats. However, pretreating the rats with different doses of FUC (500, 200, and 100 mg/kg) decreased the liver enzymes. When comparing the groups treated with fucoxanthin and the group treated with NAC to the positive controls, a significant improvement in liver functions was observed (*p* < 0.001) (Figure 1).

**Figure 2:**
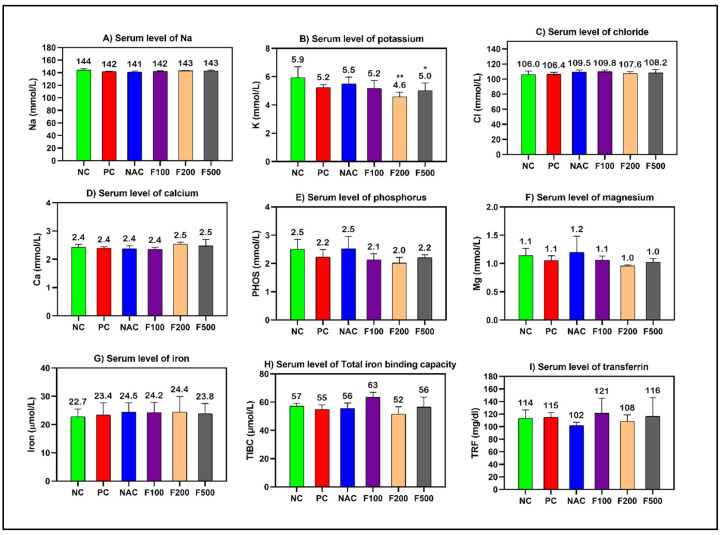
Effects of FUC on the serum levels of (**A**) sodium, (**B**) potassium, (**C**) chloride, (**D**) calcium, (**E**) phosphorus, (**F**) magnesium, (**G**) iron, (**H**) total iron-binding capacity, and (**I**) transferrin in the rats with toxicity induced by PAC. The treated groups with different doses of FUC (F100 = 100 mg/kg, F200 = 200 mg/kg, and F500 = 500 mg/kg) and NAC groups were compared to the rats with liver injury or positive control rats (PC) and healthy rats (NC). The data are expressed as mean ± SD of the fold change. * and ** indicate *p* < 0.05, *p* < 0.01, comparing all rat groups to healthy, non-treated, negative control (NC) rats.

However, non-significant changes in the levels of cholesterol, creatinine, and blood urea nitrogen (BUN) were observed. The blood glucose level was significantly increased in the PC (*p* < 0.001), NAC (*p* < 0.05), and F100 (*p* < 0.05) groups compared to NC (Figure 1). There was no noteworthy change in the serum levels of minerals and iron parameters in the treated groups compared to the PC and NC groups (Figure 2).

### 2.2. FUC Modulates the Inflammatory and Oxidative Stress-Related Markers in the Serum of PILI

After analyzing the effects of FUC on reducing the liver enzyme levels after acute liver toxicity induction by PAC, the ELISA method was performed to elucidate the inflammatory responses in the treated rats. TNF-α, IL-1β, and IL-6 are the main regulating proinflammatory cytokines; these, along with MDA, which is often utilized as a diagnostic marker of oxidative stress, were chosen for further investigation.

**Figure 3:**
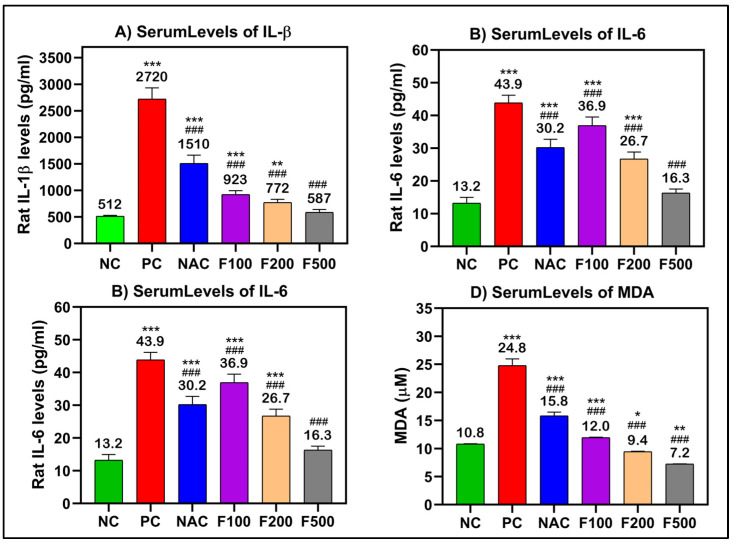
(**A**) IL-1β, (**B**) IL-6, (**C**) TNF-α, and (**D**) MDA significantly decreased after treatment of liver-injured rats with different doses of FUC. Data are expressed as the mean ± SD. One-way analysis of variance (ANOVA) was used to determine whether there were any statistically significant differences when comparing the means of the studied groups with NC and PC. *, ** and *** indicate significant difference levels of *p* < 0.05, *p* < 0.01, and *p* < 0.001 compared to NC, respectively; and ^###^ indicate significant difference levels of *p* < 0.001 compared to PC.

There were significant increases in the serum levels of TNF-α (250 pg/mL), IL-β (2720 pg/mL), IL-6 (43.9 pg/mL), and MDA (24.8 μM) in the rat groups that received paracetamol (PC) compared to the untreated healthy control group (NC). In the rat groups that had PAC liver injury after treatment with FUC, the levels of these inflammatory biomarkers were significantly decreased in a dose-dependent manner (100, 200, and 500 mg/kg). The maximum effect was observed in the groups with high doses, the F500 groups, where the levels of TNF-α (54.4 pg/mL), IL-β (587 pg/mL), IL-6 (16.3 pg/mL), and MDA (7.2 μM) were significantly decreased compared to their levels in the untreated liver injury rat group (PC) (*p* < 0.001) and NAC-treated rats (*p* < 0.001) (Figure 3).

### 2.3. FUC Modulates Inflammatory- and Oxidative Stress-Related Genes in PILI

**Figure 4:**
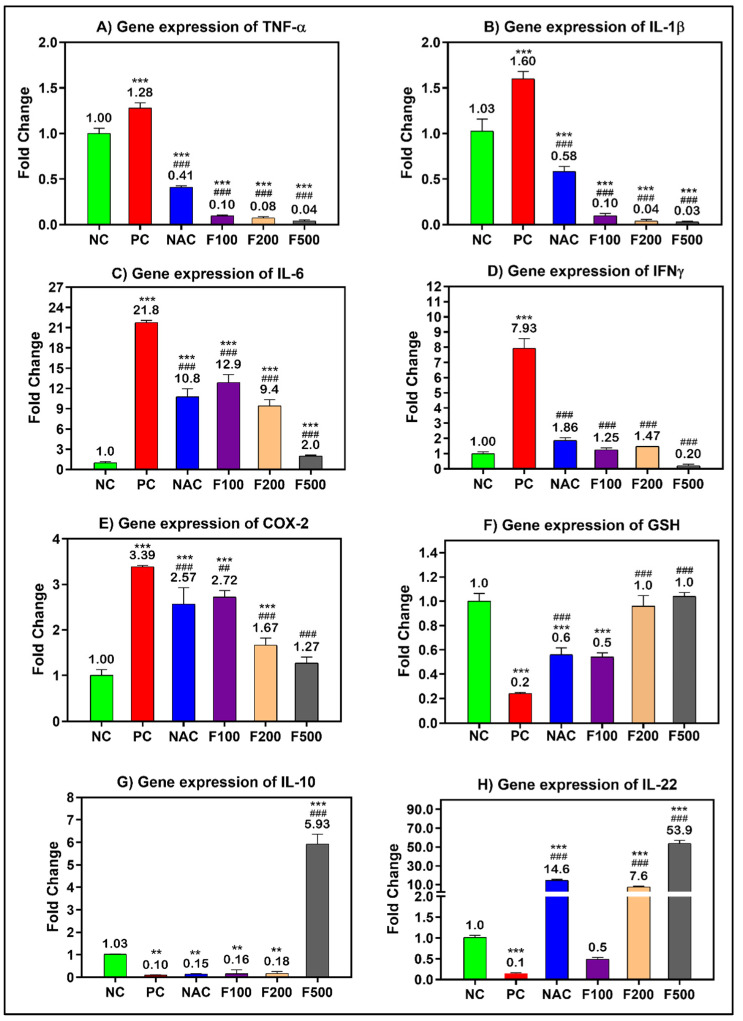
mRNA levels of the inflammatory- and oxidative stress-related genes in PILI rats with and without treatment with different doses of FUC and NAC. The groups treated with different doses of FUC (F100 = 100 mg/kg, F200 = 200 mg/kg, and F500 = 500 mg/kg) are compared to PILI or positive control rats (PC) and healthy rats (NC). The data are expressed as mean ± SD of the fold change. ** and *** indicate *p* < 0.01 and *p* < 0.001, comparing all rat groups to healthy, non-treated, negative control (NC) rats; ^###^ indicates *p* < 0.001, comparing all rat groups to the rats with liver injury or positive control rats (PC), respectively.

After confirming the anti-inflammatory and antioxidative role of FUC in the blood of liver-injured rats, the work was extended to explore the potential anti-inflammatory and antioxidative role of FUC at the tissue level. The inflammatory- and oxidative stress-related genes were significantly overexpressed in the liver tissue of rats treated with PAC; the fold change in TNF-α (1.28-fold), IL-1β (1.60-fold), IL-6 (21.8-fold), INF-γ (7.93-fold), and COX-2 (3.39-fold) compared to the untreated healthy control group (NC) is shown in Figure 4. There was a dose-dependent reduction in the expression of the above-mentioned genes in the PILI rats after treatment with F100 (100 mg/kg), F200 (200 mg/kg), and F500 (500 mg/kg). The fold change in TNF-α, IL-1β, IL-6, INF-γ, and COX-2 significantly decreased to 0.04-, 0.03-, 2.0-, 0.20-, 1.27-fold after treatment with the high dose of FUC (*p* < 0.001), respectively. Compared to NAC, FUC was more effective in the downregulation of TNF-α, IL-1β, IL-6, INF-γ, and COX-2 genes. On the other hand, the expressions of anti-inflammatory genes GSH (0.2-fold), IL10 (0.10-fold), and IL22 (0.1-fold) were significantly downregulated in the rats with PILI (PC) compared to the NC rats, as shown in Figure 4 F–H. After treatment with FUC, the expressions of GSH, IL-10, and IL-22 were significantly increased in a dose-dependent manner. The FUC dosage of 500 mg/kg was more effective in increasing their expressions to 1-fold, 5.93-fold, and 53.9-fold than in the NAC-treated rats and compared to the positive controls, as shown in Figure 4.

### 2.4. FUC Modulates Inflammatory- and Oxidative Stress-Related Proteins in PILI

**Figure 5:**
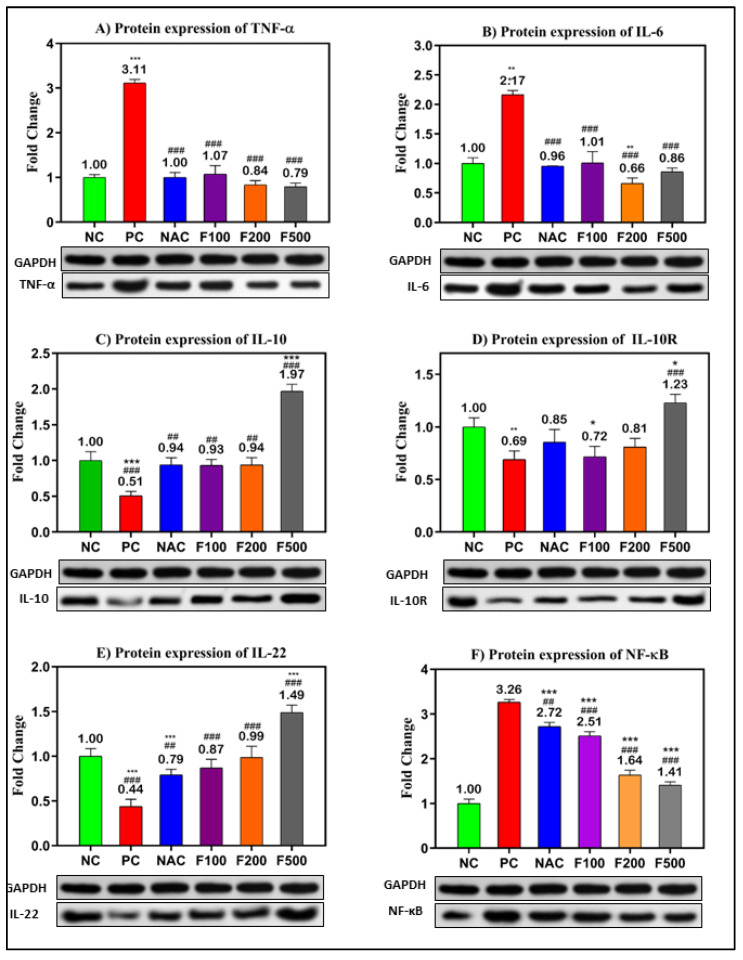
Protein levels of the inflammatory- and oxidative stress-related genes in PILI rats with and without treatment with different doses of FUC and NAC. The groups treated with different doses of FUC (F100 = 100 mg/kg, F200 = 200 mg/kg, and F500 = 500 mg/kg) were compared to the rats with liver injury or positive control rats (PC) and healthy rats (NC). The data are expressed as mean ± SD of the relative fold change in the target protein related to GAPDH as a housekeeping protein. *, **, *** indicate *p* < 0.05, *p* < 0.01, and *p* < 0.001, comparing all rat groups to healthy or negative control (NC) rats; ^##^, ^###^ indicate *p* < 0.01 and *p* < 0.001, comparing to the rats with liver injury or positive control rats (PC), respectively.

In order to confirm the previous results, the protein levels in PAC-induced liver damage were investigated. The WB technique was used to evaluate the relative expression of the selected targeted proteins (TNF-α, IL-6, IL-10, IL-10R, IL-22, and NF-кB) relative to the expression of GAPDH as a housekeeping protein (Figure S1). The inflammatory- and oxidative stress-related proteins were significantly overexpressed in the liver tissue of rats treated with PAC. The fold changes in TNF-α (3.11-fold), IL-6 (2.17-fold), and NF-кB (3.26-fold) compared to the untreated negative control (NC) are shown in Figure 5A,B,E. However, downregulations of IL-10 (0.51-fold, *p* < 0.001), IL-10R (0.69-fold, *p* < 0.01), and IL-22 (0.44-fold, *p* < 0.001) were observed in the PC rats compared to the NC rats. The treatment with different doses of FUC (100, 200, and 500 mg/kg) significantly decreased the expressions of TNF-α, IL-6, and NF-кB in a dose-dependent manner compared to the NC and PC rats, as shown in Figure 5A,B,E.

Moreover, the expressions of IL-10 (up 0.9-fold), IL-10R (up to 0.7-fold), and IL-22 (up 0.8-fold) were significantly increased after treatment with different doses of FUC. The highest dose of FUC (F500 mg/kg) showed a stronger effect on the modulation of the selected proteins than NAC, a known antidote of PAC (Figure 5).

### 2.5. FUC Modulates the Inflammatory- and Oxidative Stress-Related Proteins in PILI Tissue

**Figure 6:**
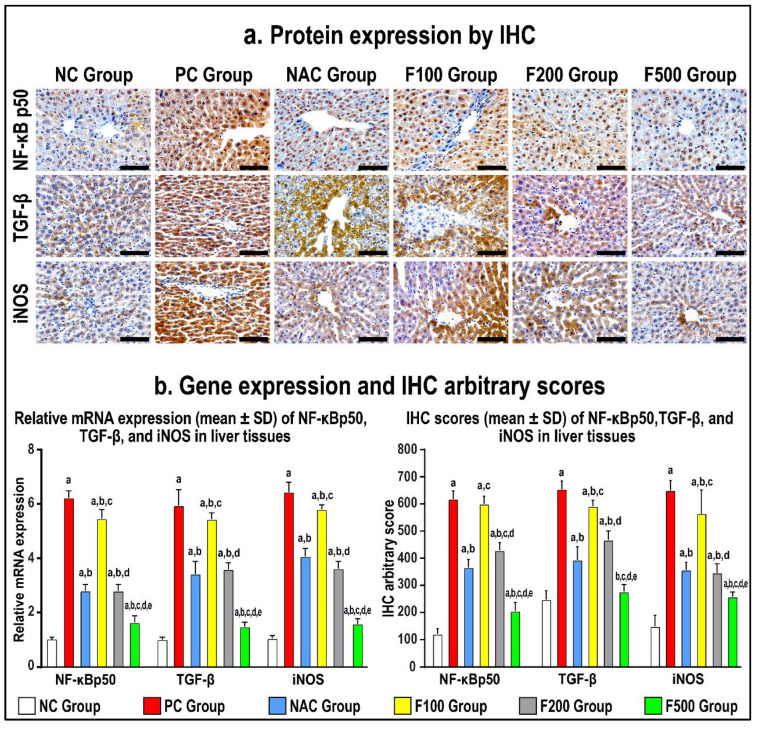
Immunohistochemistry (IHC) localization of NF-кB p50, TGF-β, and iNOS (40× objective; scale bar = 10 mm) alongside liver tissues in untreated acute liver toxicity with paracetamol (PC) and rats treated with different doses of FUC (100 mg/kg, 200 mg/kg, and 500 mg/kg) and NAC in comparison to normal control (NC) rats (**a**). (**b**) The relative mRNA expression and IHC scores in the different study groups are displayed as graph bars (data are shown as mean ± SD; a: *p* < 0.05 compared with the PC group; b: *p* < 0.05 compared with the NAC group; c: *p* < 0.05 compared with the F100 group; d: *p* < 0.05 compared with the F200 group; and e: *p* < 0.05 compared with the F500 group).

In order to confirm the previous findings, immunohistochemistry staining of the liver tissue inflammatory and oxidative markers was performed. The protein expression of NF-κB p50, TGF-β1, and iNOS increased in the PC hepatic tissues compared to the NC specimens, as shown in Figure 6 (*p* < 0.01 for all). The NAC treatment markedly reduced the protein levels of NF-κB p50, TGF-β1, and iNOS (Figure 6; *p* < 0.01 for all) relative to the PC group. However, the results of all molecules in the NAC group were significantly more abnormal than the NC group.

While there was no reduction in the gene and protein expression of NF-κB p50, TGF-β1, and iNOS in the F100 group compared to the PC group, both the F200 and F500 groups revealed markedly lower hepatic protein expression of NF-κB p50, TGF-β1, and iNOS (Figure 6). Moreover, the protein levels of the targeted molecules were considerably lower in the F100 group than the F200 group, although the F500 group showed the lowest significant gene and protein levels of NF-кB p50, TGF-β, and iNOS compared with the NC, PC, and NAC groups (Figure 6).

## 3. Discussion

During a liver function test, the concentrations of several enzymes and proteins present in the bloodstream are assessed. Elevated or reduced levels may indicate hepatic dysfunction. Both aspartate transaminase (AST) and alanine transaminase (ALT) are soluble enzymes found in the cytoplasm of hepatocytes [23]. After hepatocyte damage, the membrane permeability increases, allowing ALT and AST to penetrate the bloodstream. Consequently, serum ALT and AST activities can indicate the severity of hepatocyte injury. Conventionally, serum ALT and AST activities are used as sensitive markers to assess liver injury [24]. Additionally, it is worth noting that both the liver and bones possess an enzyme called alkaline phosphatase (ALP), which plays a crucial role in the elimination of phosphate groups. Elevated levels of alkaline phosphatase may indicate hepatic impairment or pathology, such as obstructed bile ducts or specific skeletal abnormalities. The present study observed elevated levels of ALT, AST, and ALP in the PCA model group (PC) compared to the control group (NC), suggesting that the rats in the model group successfully developed PAC-induced liver injury. Furthermore, it was found that FUC demonstrated protective benefits against PCA-induced toxicity. The activities of ALT and AST in the rats belonging to the NAC group and FUC groups (F100, F200, and F500) exhibited a statistically significant decrease compared to the rats in the PC group, and approached the levels observed in the rats in the NC group over time (refer to Figure 1). The aforementioned findings provide additional evidence for this result [25,26], indicating that FUC can regulate ALT and AST activities, thereby reducing PAC-induced liver injury. Very few published studies have examined the effects of fucoxanthin on patients with liver injury [27,28,29]. A randomized, placebo-controlled clinical trial with a 24-week follow-up was previously conducted on 42 patients [30]. In the treatment group, three capsules of low-molecular-weight fucoidan and high-stability fucoxanthin were administered twice daily. The ratio of ALT/AST (alanine aminotransferase/aspartate aminotransferase) was proposed as a diagnostic alternative for hepatic steatosis [30]. After 6 months, serum AST/ALT levels were lower in the FUC treatment group compared to the placebo group, indicating a decrease in hepatotoxicity in NAFLD patients. At three and six months, the expression of the cytokines IL-6 and IFN-γ were reduced in the FUC group compared to the placebo group, reducing inflammation in patients with NAFLD [30]. However, there was no significant difference in the lipid profile between treated and untreated rats because acute liver injury requires more time to observe the change in these parameters in the lipid profile, which is in agreement with our findings (Figure 1).

In addition to liver enzymes, the measurement of protein levels in the bloodstream can be utilized as a reliable biomarker for evaluating liver function. This study observed a notable upregulation of inflammatory- and oxidative stress-related markers in both hepatic tissue and blood samples of rats treated with PAC, as compared to the control group, at both the gene and protein levels. In contrast, the rats given PILI (PC) exhibited a significant downregulation of the expression of the anti-inflammatory genes IL10 and IL22 when compared to the negative control group.

In this study, RT-PCR, ELISA, WB, and/or IH were applied to evaluate this effect on key markers such as TNF-α, IL-1β, IL-6, NF-кB, INF-γ, iNOS, TGF-β, IL10, and IL22 in the rats with PAC-induced liver injury (PC) compared to negative controls. There was a dose-dependent modulation in the expression of the above-mentioned genes and proteins in the rats with PAC-induced liver injury after treatment with F100 (100 mg/kg), F200 (200 mg/kg), and F500 (500 mg/kg). Previously, it was reported that liver inflammation is a sign of almost all liver disease and a major contributor to liver damage, and PAC toxicity causes damage to the liver’s central lobule region, which results in the release of reactive oxygen species (ROS) and the generation of proinflammatory cytokines [1]. The biological system includes two categories of free radicals: reactive oxygen species (ROS) that are derived from O_2_ metabolism and reactive nitrogen species (RNS) that are derived from nitric oxide and superoxide [31]. ROS include oxygen free radicals such as superoxide, hydroxyl radicals, and peroxyl radicals as well as non-radical reactive oxygen species such as hydrogen peroxide, hypochlorous acid, and ozone. Inducible nitric oxide synthase (iNOS) and nicotinamide adenine dinucleotide phosphate (NADPH) oxidase are the enzymes responsible for producing RNS, which include nitrogen-based radicals and non-radicals, such as nitrogen dioxide, nitric oxide radicals, and peroxynitrite [31]. Therefore, the chief contributors to oxidative stress induced by a PAC overdose are mitochondrial superoxide and peroxynitrite [17]. Superoxide reacts with nitric oxide to generate the highly reactive peroxynitrite species, which is the primary source of oxidative and nitrosative stress [17].

The induction of oxidative stress by PAC is a crucial factor in the pathogenesis of acute ALD. This can increase the level of oxidative stress in many tissues, particularly the liver. Antioxidants protect cells from reactive oxygen species (ROS), but excess lipid peroxides, which can cause liver injury [32], are able to quickly neutralize antioxidant activity. The effect of FUC on malonaldehyde (MDA) levels in rodents was shown in this study. The activities of MDA in the PC group were significantly greater than those in the NC group, whereas the MDA content decreased significantly after treatment with FUC. MDA may, therefore, be a by-product of free radicals acting on lipid peroxidation. MDA content, a common indicator of membrane lipid peroxidation, reflects the degree of lipid peroxidation within the body [33]. This suggests that FUC may effectively prevent the excessive oxidation of hepatocytes caused by PAC consumption and could increase the body’s antioxidant capacity, thereby providing substantial protection against PAC-induced liver damage. Consistent with the findings of this study, Zheng et al. (2019) reported that FUC protected mice from alcohol-induced liver damage [26].

The various observations about the involvement of inflammation in liver injury caused by PAC are primarily attributed to the activation of NF-кB and the subsequent induction of inflammatory cytokines in areas adjacent to or distal from innate sensing mechanisms. This assertion holds special validity in the case of prototypic interleukin-1 (IL-1), interleukin-1 receptor antagonist (IL-1RA), tumor necrosis factor-alpha (TNF-α), and interleukin-6 (IL-6) [34], all of which are generated during PILI [32]. NF-кB is the most important transcriptional regulator in the inflammatory signal transduction pathway, and it plays a key regulatory role in the transcriptional production of the pathway’s inflammatory mediators [35]. Under normal conditions, NF-кB binds to its inhibitor IкB when both are dormant. This makes IкB phosphorylate and break down. Then, NF-B is turned on and transfers into the nucleus, which leads to the production and release of cytokines that cause inflammation. The excessive accumulation of lipids following toxic consumption has been linked to liver cell injury, inflammation, the upregulation of proinflammatory markers, and liver inflammation. Kupffer cells are activated and the immune system of the liver increases when a large amount of toxic substance is broken down [35,36]. Rapid activation of transcriptional regulators like NF-кB by activated Kupffer cells results in the production of high levels of inflammatory factors such as TNF-α, IL-1β, IL-6, and IFN-γ, as well as other inflammatory mediators, which can exacerbate inflammatory infiltration and injury to liver tissue, ultimately causing liver inflammation [37,38]. To investigate the anti-inflammatory mechanism of FUC on the protective effects of PAC-induced liver injury, ELISA, RT-PCR, WB, and IH were used to analyze the concentrations of NF-кB-induced signaling pathway-related genes and proteins. As shown in Figure 3, Figure 4, Figure 5 and Figure 6 after PAC induction, the concentrations of NF-кB were significantly increased, and a downstream signal cascade was initiated, leading to the significant upregulation of TNF-α, IL1β, and IL-6. However, the FUC pretreatment groups had significantly decreased expression of downstream proteins than the PC group after PAC administration. Overall, rthe esults indicate that FUC can attenuate PAC-induced hepatic inflammatory responses by inhibiting NF-кB-induced signaling pathways.

We evaluated the expression of associated inflammatory factors to determine if FUC might reduce the inflammatory response produced by PAC-induced liver damage. Figure 3, Figure 4, Figure 5 and Figure 6 show an increase in the levels of proinflammatory factors (TNF-α, IL-1, IL-6, NF-кB, INF-γ, and iNOS) produced from liver tissue after PAC induction. This suggests that these cytokines play a role in the development of PAC-induced liver injury in rats. When FUC was given to the rats, there was a dose-dependent decrease in the expression of several molecules in the liver tissue. This shows that the inflammatory response based on PAC-induced liver injury can be decreased by FUC’s ability to effectively regulate the release of proinflammatory cytokines.

Previously, FUC decreased the levels and the expression of inflammatory response molecules in liver injury and was able protect the liver from damage by alcohol-induced liver injury [26]. These data indicate that FUC can effectively control the secretion of proinflammatory factors, thereby reducing the inflammatory response caused by alcohol-induced liver injury.

## 4. Materials and Methods

### 4.1. Chemicals

The fucoxanthin and paracetamol (98%) were purchased from Sigma-Aldrich Chemie GmbH (Taufkirchen, Germany). FUC is characterized by its general formula C42H58O6, CAS Number 3351-86-8, and molecular weight of 658.91. The purity of fucoxanthin is stated to be equal to or more than 95.0%, as determined by high-performance liquid chromatography (HPLC) area percentage.

### 4.2. Study Design and Treatment Protocols

This case–control study included 30 male Wistar rats, each weighing 200–250 g at 12 weeks old, which were housed in clean, sterile polyvinyl cages (five rats per cage) maintained at a constant temperature of 22–24 °C and under a 12-h darkness/light cycle. The rats were maintained on a standard laboratory pellet diet and provided with free access to water.

**Figure 7:**
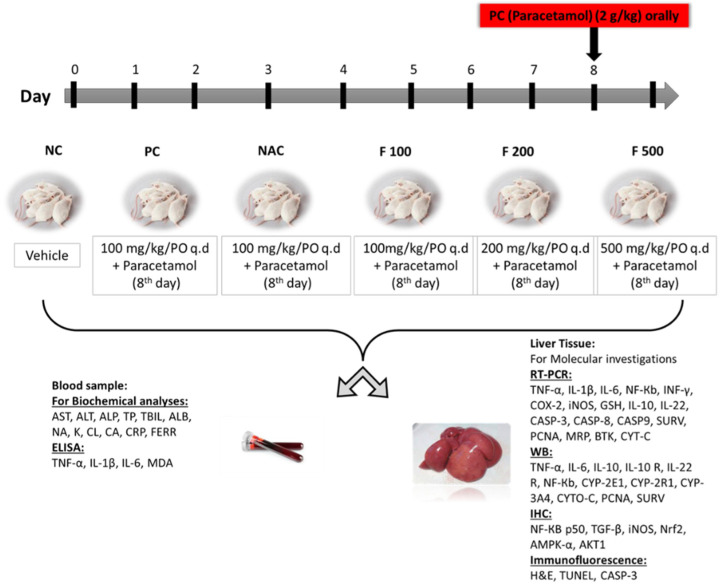
The study design, classification of animals into 6 groups, sample collection, techniques used, and biochemical and molecular markers evaluated.

The animals were classified into 6 groups equally and randomly for the duration of the study (9 days), as previously reported [39]. *NC group*: the first group served as a negative control for untreated healthy rats (NC) and was given a dissolved vehicle. *PC group*: the second group was the positive control group (PC), which on day 8 was given 1.5 mL of 2 g/kg PAC orally. *NAC group*: the third group was administered 1200 mg/kg of N-acetyl cysteine once daily from days 1 to 7 then orally administered 1.5 mL of 2 g/kg of PAC on day 8. *F100 group*: the fourth group was administered 100 mg/kg of FUC once daily from days 1 to 7 then orally administered 1.5 mL of 2 g/kg of PAC on day 8. *F200 group*: the fifth group was administered 200 mg/kg of FUC once daily from days 1 to 7 then orally administered 1.5 mL of 2g/kg of PAC on day 8. *F500 group*: the sixth group was administered 500 mg/kg of FUC once daily from days 1 to 7 then orally administered 1.5 mL of 2 g/kg of PAC on day 8. The study ended after nine days. Throughout the study, all animals had unrestricted access to food and water. Following an overnight fast, all groups were euthanized on day 9; diethyl ether (Fisher Scientific, Loughborough, UK) was used to sedate the rats before slaughter and collecting samples (Figure 7).

### 4.3. Collection of Samples

The blood samples were taken in a plain container and the sera were separated. Each animal’s liver was removed, and a section of it was prepared for paraffin embedding. To measure biochemical parameters, another portion was digested with 6:1 perchloric acid using a microwave digestion system. Another portion of liver tissue was homogenized in RIPA lysis buffer with protease inhibitors (Santa-Cruz Inc., Santa-Cruz, CA, USA) for total protein extraction, and the protein concentrations were assessed using the PierceTM Rapid Gold BCA Protein Assay Kit (Thermo Fisher Scientific, Waltham, MA, USA). Finally, using the Paris kit, RNA was obtained from hepatic tissues (Thermo Fisher Scientific). The quantified RNA concentrations were determined using a Jenway Genova Nano Micro-Volume Life Science Spectrophotometer, DNA/RNA/Oligo/Protein; 115-230 VAC.

### 4.4. Biochemical Parameter Measurement

Using an automated chemistry analyzer (Humanstar, Co., Schindlerhof, Germany), in accordance with the manufacturer’s instructions, the serum liver markers, including aspartate aminotransferase (AST), alanine aminotransferase (ALT), alkaline phosphatase (ALP), total protein, total bilirubin, albumin, Na, K, Cl, Ca, CRP, ferritin, etc., were measured.

### 4.5. mRNA Isolation

The required amount of lysis buffer was supplemented with β-mercaptoethanol or DTT. Then, 10 µL of 14.3 M β-mercaptoethanol or 2 M DTT was added to each 1 mL volume of lysis buffer required. Liver samples were collected, and RNA isolation and purification were carried out on the same day. According to the manufacturer’s instructions, the purified RNA was used for downstream applications or stored at −20 °C or −70 °C until use.

RNA samples were quantified by passing the solution many times through a pipette tip after being resuspended in RNase-free water. Total RNA concentration was determined using the Jenway Genova Nano Micro-Volume Life Science Spectrophotometer. The ratio of absorbance at 260/280 nm and 260/230 nm was utilized to determine the purity of the RNA. RNA with a 260/280 ratio of roughly 2.0 was considered “pure”.

### 4.6. cDNA Synthesis

Following the manufacturer’s instructions, cDNA was synthesized from 1 mg of RNA using the Precision qScript Reverse Transcription kit (Primer Design) and oligo-dT primers (Table 1). The cDNA was diluted (1:10) and 5 mL was used for each reaction; each experiment was conducted in triplicate. The protocol was carried out according to the manufacturer’s protocol.

### 4.7. Gene Expression Using RT-PCR

The relative expression of the targeted genes (Table 1) in the different study groups was measured using real-time PCR. Using a high-capacity reverse transcription kit from Thermo Fisher Scientific and following the manufacturer’s procedure, total RNA was reverse-transcribed into cDNA. An Applied Biosystems 7500 Fast Dx Real-Time PCR Instrument (Thermo Fisher Scientific) was used to conduct the RT-PCR in a 96-well configuration, and the results were assessed using a relative standard curve method of 40 cycles of reaction amplification (95 °C for 15 s and 60 °C for 1 min), as previously described [22].

### 4.8. Gene Expression Calculation

As detailed before [32], a post-PCR melting curve analysis was conducted to establish the specificity of the primers. Ct values of the samples were calculated in comparison to the NC group; the gene expression data were expressed as a fold change 2^−ΔΔCt^ and normalized to GAPDH as a housekeeping gene.

### 4.9. Liver Tissue Protein Isolation and Quantification

About 4 g of liver tissue was taken in an OMNI bead tube (Omni Bead Ruptor-12) according to the manufacturer’s instructions. RIPA lysis buffer with protease inhibitors was used to extract the total amount of tissue proteins (Santa Cruz Biotechnology Inc., Santa Cruz, CA, USA). Centrifugation with a relative centrifugal force (RCF) of 1681.1× *g* for 10 min at 4 °C was used to separate the cells from the tissue homogenates. The supernatant was then stored at −80 °C.

### 4.10. Immunoblotting

Proteins were transferred onto a nitrocellulose blotting membrane (Bio-Rad, Hercules, CA, USA) by immersing cassettes in a tank filled with blotting/transfer buffer (3.03 g of trizma base, 14.4 g of glycine, 0.375 g of SDS, 200 mL of MeOH, and 800 mL of dH_2_O) and left overnight at 25 V. The blots were washed for 5 min with TBST (6.6 g of NaCl, 25 mL of 1 M Tris HCl pH 7.5, and 0.05% Tween 20 added to 1L of dH_2_O). The membranes were immersed in blocking buffer with shaking for 1 h at room temperature (10% *w*/*v* Marvel dry skimmed milk in TBST) to block the non-specific binding of proteins. The membranes were washed 2× in TBST for 5 min with shaking.

Polyclonal rabbit antibodies were employed as the primary antibodies for TNF-α, IL-6, IL-10, IL-10 R, IL-22 R, and NF-Кb (Santa-Cruz Biotechnology Inc) were added 1 at a time to detect proteins at concentrations of 1:1000 or 1:2000, respectively, in 10 mL of TBST containing 1% skimmed milk in a tube rolling at room temperature for 2 h, then overnight at 4 °C. The membranes were washed in TBST (3×, 5 min each) to remove excess unbound primary antibodies. TBST (10 mL) containing 1% skimmed milk and 1:5000 or 1:2400 secondary antibody (polyclonal swine anti-rabbit immunoglobulin or goat anti-mouse), respectively, was added to the membrane and was foiled and shaken for 1 h at room temperature. The membranes were washed 4× in TBST (5 min each) to remove excess unbound secondary antibodies and the remaining procedure was conducted as previously described [Althubiti, 2020 #3249].

### 4.11. Enhanced Chemiluminescence Detection

A mixture of equal volumes of detection reagents A and B (Enhanced chemiluminescence kit solution, Thermo-Scientific, USA) was spread on the membrane blot for 1 min. The membrane was transferred to a developing cassette, attached to a chemiluminescence X-ray film (Thermo-Scientific, USA), and was kept closed for 15 min. The membrane was washed in developer reagent under red light, and when protein signals on the X-ray film were strong enough, it was washed with running H_2_O and immersed in fixing reagent for 1 min, before it was washed and dried.

### 4.12. Enzyme-Linked Immunosorbent Assay

Special rat ELISA kits were used to measure the serum levels of TNF-α, IL-1β, and IL-6 (Cloud-Clone Corp., Houston, TX, USA), and malonaldehyde (MDA) (Abcam Chemical Co., Cambridge, UK) was used according to the manufacturer’s instructions.

### 4.13. Immunohistochemistry

Santa-Cruz Biotechnology Inc.’s goat and rabbit primary polyclonal IgG antibodies against NF-КB p50, TGF-β, iNOS, Nrf2, AMPK-α, and AKT1 were applied. The Elite Vectastain Rabbit or Goat ABC kits from Vector Laboratories Inc., Newark, CA, USA, were used to stain the liver tissues in accordance with the manufacturer’s instructions. Each primary antibody was diluted 1:200 and left to stand at room temperature for 3 h. Two blinded examiners used an EVOS XL Core microscope with a 40× objective lens to examine the samples and evaluate the immunostaining. Each protein’s picture was selected randomly from 15 non-overlapping fields/sections.

### 4.14. Statistical Analysis

The statistical analysis and drawing of the figure were carried out using GraphPad Prism version 9, and the results were expressed as mean standard deviation (SD). The Kolmogorov and Smirnoff test and the Levene test were used to determine the results’ normality and homogeneity. One-way ANOVA and the LSD test were also used to compare the groups. The *p*-value cutoff for significance was chosen to be <0.05.

### 4.15. Ethical Approval

The study was approved by the Biomedical Research Ethics Committee, Faculty of Medicine, Umm Al-Qura University (HAPO-02K-012-2023-11-1873), and the experiments were in accordance with the European guidelines for the care and use of laboratory animals.

## 5. Conclusions

Our findings showed that FUC is an effective substance for preventing PAC liver injury. This study demonstrates that FUC inhibits PAC-induced oxidative stress by decreasing the concentration of oxidative products and elevating iNO-mediated antioxidant responses. In addition, FUC prevents hepatic inflammation by inhibiting signaling pathways induced by NF-кB. These findings suggest that FUC has great potential for the development of healthy diets that prevent PAC liver injury. Further studies are required to determine the ideal dose of FUC for preventing PAC liver injury and its pharmacokinetic and pharmacodynamic effects in this model. However, the findings of this study suggest that FUC is a promising candidate for the development of new preventive strategies.

## Figures and Tables

**Table 1:** Primers used for real-time RT-PCR.

Gene	Sequences
**GAPDH**	F: AGGTCGGTGTGAACGGATTTG R: TGTAGACCATGTAGTTGAGGTCA
**TNF-α**	F: CTCTTCTGCCTGCTGCACTTTG R: ATGGGCTACAGGCTTGTCACTC
**IL-1β**	F: GCCCTTTTGCTTCAGGGTTT R: TCCAATGTCCAGCCCATGAT
**IL-6**	F: TTGACAGCCACTGCCTTCCC R: CGGAACTCCAGAAGACCAGAGC
**NF-кB**	F: CCTCTACACATAGCGGCTGG R: GCACCTTGGGATGCGTTTTT
**INF-γ**	F: TGCAACCCTCCTAGACTCATTCT R: CCAGCAGGGCGTCTTCCT
**COX**	F: GGTTCACCCGAGGACTGGGC R: CGCAGGTGCTCAGGGACGTG
**iNOS**	F: GAAGCGGAGACCCAAGAGA R: TCGCAAAGAGGATGGTGACT
**GSH**	F: GAAATCCCAGCGCCCTA R: CACTCACCTTCGACTTCTCTTGCT
**IL-10**	F: CCACATGCTCCGAGAGCTGA R: TCTTCACCTGCTCCACTGCC
**IL-22**	F: GCAGGCTTGACAAGTCCAACT R: GCCTCCTTAGCCAGCATGAA

## Data Availability

All data is available in the article.

## Supplementary Materials

The following supporting information can be downloaded at: https://www.mdpi.com/article/10.3390/md21110592/s1, Figure S1: Final WB Suppl Figure.
