# Supplementary material for: Antioxidative and Anti-Inflammatory Protective Effects of Fucoxanthin against Paracetamol-Induced Hepatotoxicity in Rats
Source: Mar Drugs. 2023 Nov 14;21(11):592. doi: 10.3390/md21110592 (PMC10672227; doi:10.3390/md21110592)

## TNF-α protein Expression (17 KD)

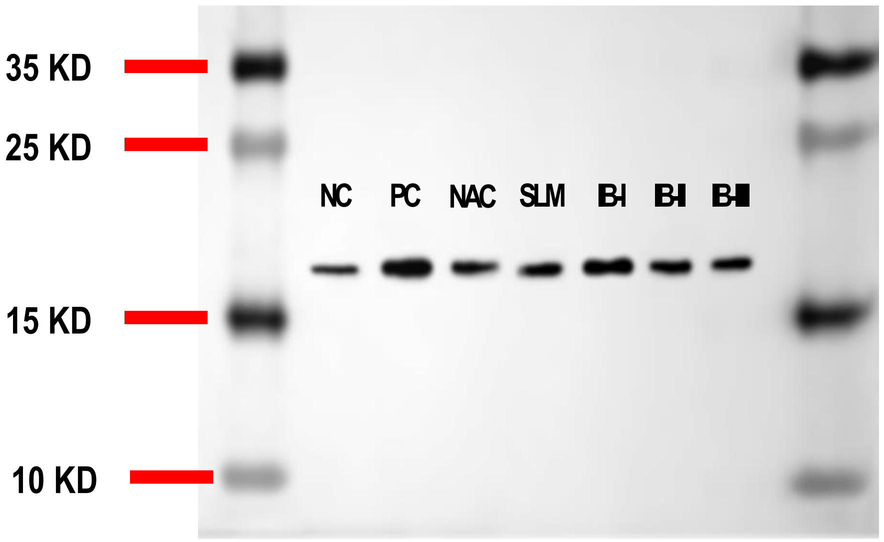


## IL-10 protein Expression (17 KD)

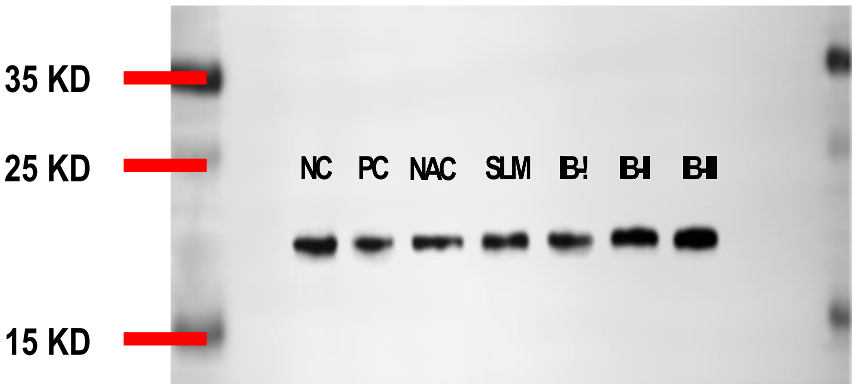

## IL-22 protein Expression (17 KD)

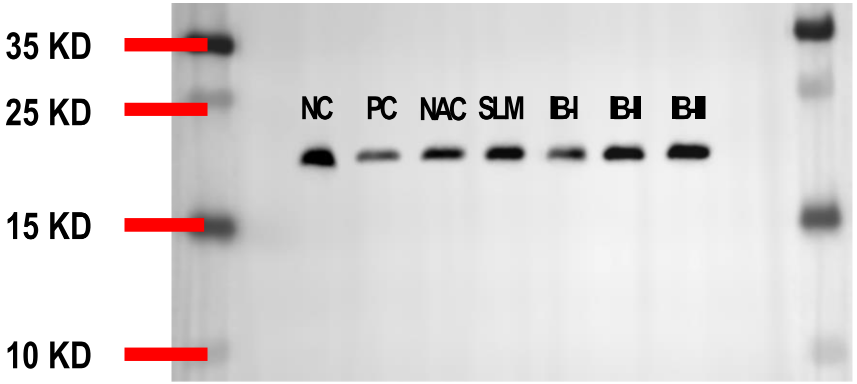


## CYP2E1 protein Expression (57 KD)

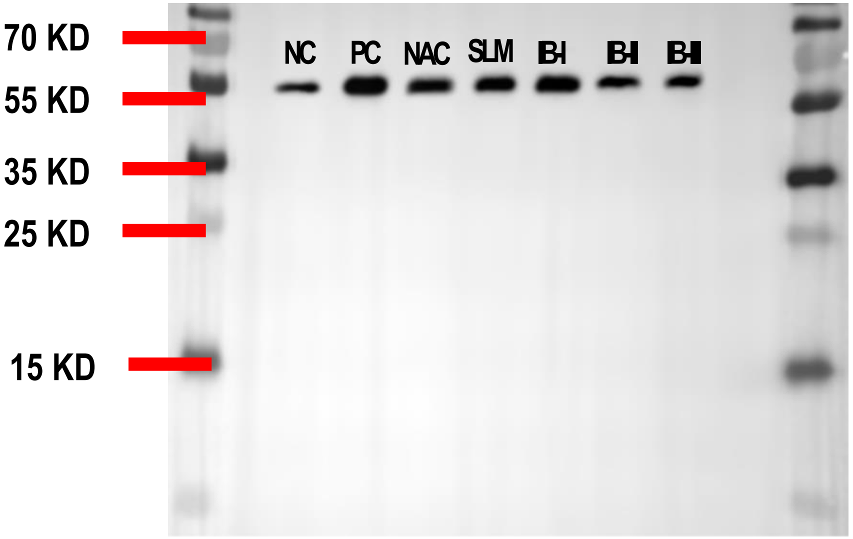

CYP3A4 protein Expression (57 KD)

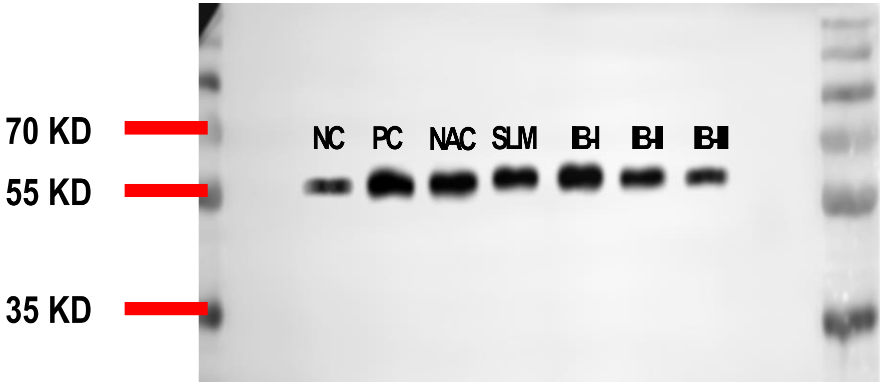


BCL2 protein Expression (26 KD)

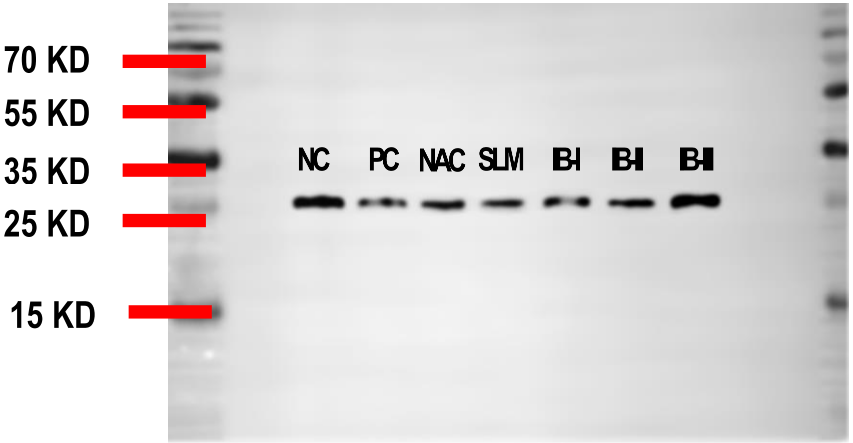

## PCNA protein Expression (36 KD)

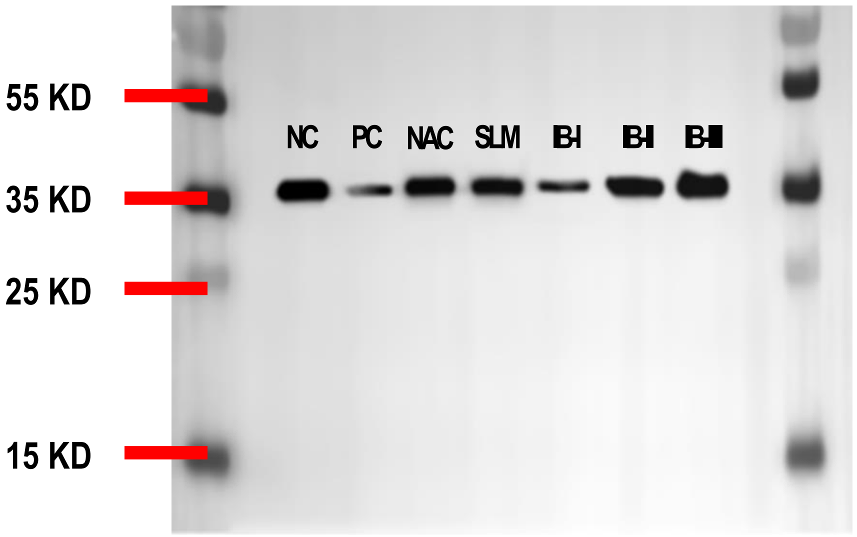


## Casp-3 protein Expression (17 KD)

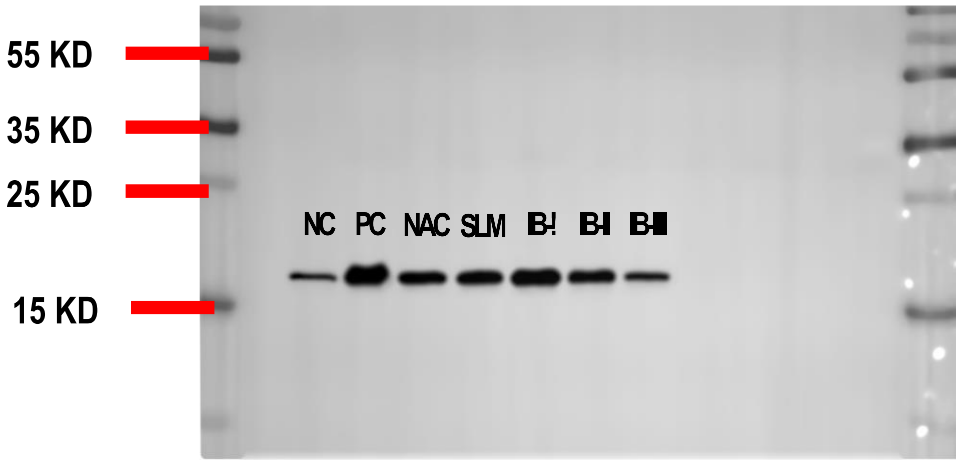

# GAPDH protein Expression (37 KD)

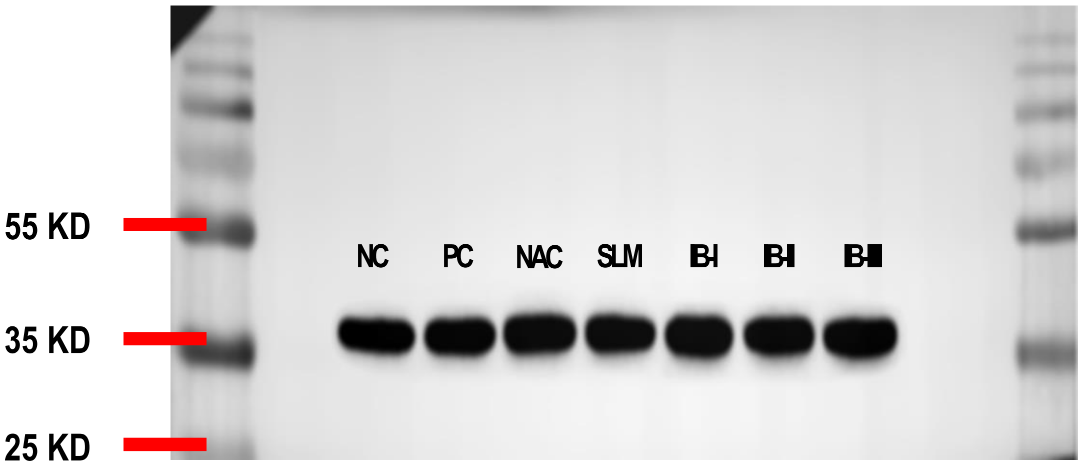

Supplement: Supplementary file 1 [file marinedrugs-21-00592-s001.zip › marinedrugs-2654917-supplementary.pdf]
